# Understanding the risk of metastasis in lower lip carcinoma: clinical insights and prognostic implications

**DOI:** 10.1186/s13005-025-00576-9

**Published:** 2026-02-03

**Authors:** Katharina Theresa Obermeier, Ella Bachmann, Ina Dewenter, Yoana Malenova, Philipp Poxleitner, Paris Liokatis, Wenko Smolka

**Affiliations:** 1 Department of Oral and Maxillofacial Surgery and Facial Plastic Surgery, University Hospital, LMU Munich, Lindwurmstrasse 2a, Munich, 80337 Germany; 2 Department of Oral and Maxillofacial Surgery, University Hospital Augsburg, Sauerbruchstraße 6, Augsburg, 86179 Germany

**Keywords:** Cutaneous squamous cell carcinoma, Lower lip, Metastases, Grading

## Abstract

**Backgrounds:**

Cutaneous squamous cell carcinoma accounts for the second most skin tumor and is mostly found in the head and neck area. Lymph nodal spreading appears in around 5% of all patients and worsens the prognosis as well as overall survival. Especially the tumor localisation in the lower lip is known for a higher risk of lymph nodal metastases.

**Methods:**

Data of 71 patients suffering from cutaneous squamous cell carcinoma (CSCC) of the lower lip were analyzed retrospectively. Risk factors such as tumor size and depth of invasion and other histopathological findings were collected. Logistic regression analysis was performed to determine the value of different risk factors.

**Results:**

One-third of all patients suffered from lymph nodal metastases. Poor differentiation was the strongest risk factor for lymph nodal spreading. Tumor diameter of more than 1.6 cm and depth of invasion greater than 0.85 cm were also considered as factors. Patients with G3 tumors had a 19.1 times higher risk for lymph nodal spreading.

**Conclusion:**

The lower lip location has a high risk for lymph node metastasis in CSCC. Overall, poor differentiation was the strongest factor associated with an increased risk of lymph node metastases in this patient cohort.

## Introduction

Cutaneous squamous cell carcinoma (CSCC) accounts for the second most common type of skin cancer worldwide [1]. Sun exposure, immunosuppressive therapy, and fair skin are the main risk factors for developing CSCC [2]. CSCC is primarily located in the head and neck area; common localizations are the ear, nose, eye, lip, cheek, and scalp [3]. Over the past few years, the incidence rate increased by 50% to 300%, depending on the geographic region [4, 5].

The prognosis of CSCC is generally good, with high rates of local control and cure rates of 95% after surgery [6]. However, once nodal spread occurs, prognosis deteriorates markedly, with a mean survival of approximately two years [7, 8].

Around 5% of the patients with CSCC develop metastases in the cervical lymph nodes [6]. Prognostic factors are surgical margins, immunosuppression, age, depth of invasion, number of affected lymph nodes, and staging [9]. The histopathological desmoplastic subtype is also associated with an increased risk of metastatic spreading [10]. Especially CSCC of the lower lip is a challenge in treatment, and it is known for a higher risk for lymph nodal spreading and death compared to other localisations of CSCC [10–12].

This study aims to determine the significance of various risk factors for lymph nodal metastasis of CSCC of the lower lip, as well as the preferred neck levels for nodal spreading.

## Materials and methods

This retrospective study was approved by the institutional review board of the University Hospital of Munich, Germany (Munich, Germany; UE Nr 22–0089). The present retrospective study includes 71 patients treated as inpatients in our hospital between 2007 and 2023. Demographic data, tumor localization, size and depth of tumor invasion, date of primary diagnosis, time to recurrence, number of simultaneously CSCC, simultaneously present basalcell-carcinomas, histopathological and immunohistochemical findings and average follow-up time were collected, and analyzed. Staging was performed according to the AJCC/UICC TNM classification (8th edition). Staging and follow-up imaging were performed with contrast-enhanced CT and/or MRI according to institutional protocols. All examinations were interpreted in routine clinical workflow by board-certified radiologists at our tertiary university hospital; imaging reports were abstracted from the medical record. No centralized re-reading was undertaken, and readers were not blinded to clinical data.

### Inclusion criteria

**Fig. 1:**
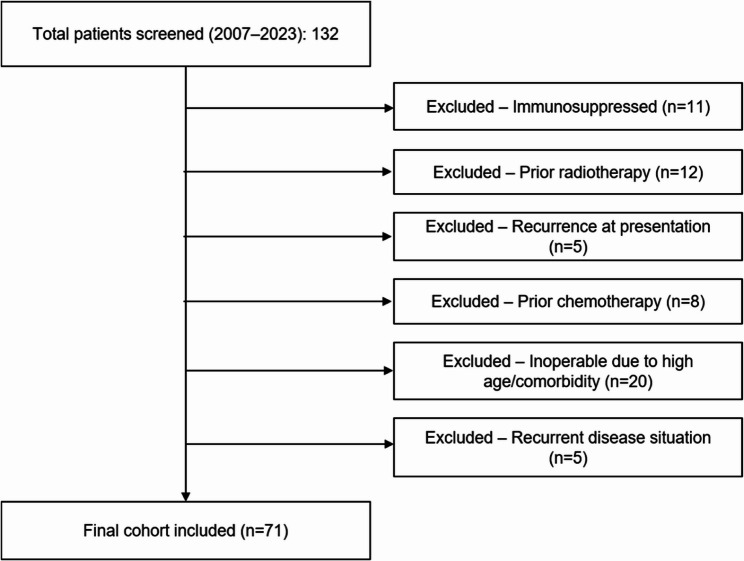
Patient selection process according to the STROBE guidelines

Patients with CSCC of the lower lip were included. Patients with primary diagnosis of CSCC of the lower lip, regular follow-up appointments, and available photo documentation were included. CT and MRI were the imaging modalities of choice for the detection of metastases and a further inclusion criteria. Figure 1 presents the STROBE flow diagram of patient inclusion and exclusion.

### Exclusion criteria

Patients who initially presented with a recurrence of CSCC were excluded. As CSCC based on immunosuppressive treatment behaves more aggressively and differently, this kind of patients were excluded from this study. Further exclusion criteria were prior radio- or chemotherapy.

### Statistical analysis

Statistical analysis was conducted using SPSS^®^ 24 version 4.0 (SPSS Inc., Chicago, IL, USA). Normally distributed data were presented using mean ± standard deviation (SD). Non-normal distributed data was illustrated by depicting median and interquartile ranges. Mann-Whitney-U-test has been performed to identify the influence of grading for lymph nodal spreading. The odds ratio (OR) for developing lymph nodal spreading in non-differentiated (G3) CSCC was calculated using cross-tables. Cut-off values were determined using ROC-analysis for the influence of tumor diameter and depth of invasion by using the Youden’s index. Afterwards, logistic regression analysis for determining the risk factors for lymph node spreading has been calculated. The final multivariable model included 7 candidate predictors with 21 events, giving an EPV ≈ 3:1. To mitigate overfitting in this low-EPV setting, we performed penalised logistic regression (ridge) and bootstrap internal validation (1,000 resamples) as sensitivity analyses. Penalised models were specified with L2 regularisation; hyper-parameters were tuned by stratified k-fold cross-validation. We report penalised odds ratios (by exponentiating coefficients) alongside the conventional logistic estimates.

Statistical significance was defined as *p* < 0.05. Localizations of metastases were reported in a descriptive style.

## Results

### Sample

The gender distribution was 33 (46.5%) to 38 (53.5%), predominately men. The mean age amounted to 77.8 years ± 11.35. In 29 patients, the tumor was staged as T1, in 20 as T2, 20 patients had T3 CSCC, and 2 patients had T4 primary tumors (UICC TNM classification). The average diameter was 1.98 cm ± 1.36 cm. The average depth of invasion was 0.64 cm ± 0.56 cm. Table 1 gives an overview of staging. Overall, 21 patients (29.6%) suffered from lymph nodal spreading. Eight of those patients showed suspicious lymph nodes in staging CT.

**Table 1:** Staging of patients with metastases

age	gender	staging	affected level
77	male	T1N1M0	Level Ib
92	female	T2N1M0	Level IIa
91	female	T2N2M0	Level Ib
79	male	T3N2M0	Level Ib
81	female	T1N1M0	Level Ia
100	male	T3N1M0	Level Ib
87	male	T3N1M0	Level Ib
60	female	T2N2M0	Level Ib both sides
88	female	T1N2M0	Level Ib and IIa
96	female	T1N2M0	Level Ib both sides
52	male	T1N1M0	Level Ib, III, IV
81	female	T3N3M1	Level Ib
92	female	T2N1M0	Level Ib both sides
58	female	T3N2M0	Level Ib, IIa, III
77	female	T4N1M0	Level Ib
61	male	T3N2M0	Level Ib, IIa, IV
91	female	T2N1M0	Level Ib
32	male	T2N1M0	Level Ib
77	female	T4N1M0	Level Ib
61	male	T3N2M0	Level II
91	female	T2N1M0	Level Ib

Local recurrence of the tumor was found in 16 patients (22.5%) and occurred after a mean period of 10 months. R1-resection was present in 5 patients (7.0%). In 3 patients re-resection was performed. Due to the proximity to the skull base, R0 resection was not possible in the other two patients. Both patients developed lymph nodal spreading. The desmoplastic type was found in 3 (4.2%) cases, and perineural invasion in 7 cases (9.8%). However, those risk factors did not significantly influence the development of lymph nodal spreading in our patients.

On histopathological grading, well-differentiated (G1) CSCC was seen in 18 patients (25.4%), moderate differentiation (G2) was found in 36 (50.7%) patients, and poor differentiation (G3) in 17 patients (23.9%). 43 (60.6%) patients received neck dissection due to advanced tumor stage or suspicious lymph nodes in preoperative computed tomography. One patient with G1-differentiation, 3 patients (4.2%) with G2-differentiation and 14 (19.7%) patients with G3-differentiation developed lymph node metastases. Overall follow-up amounted 63.3 months (range 25–192 months).

### Predicting risk factors for lymph nodal spreading

The results of the Mann-Whitney-U-Test regarding grading (G1, G2 and G3) and lymph nodal spreading showed a statistically significant result (*p* = 0.00) for lymph nodal spreading. Fisher’s exact test for comparing patients with G3 and lymph nodal spreading resulted in a *p* = 0.00. Therefore G3 tumors were associated with a greater risk for lymph nodal spreading (*p* = 0.00) and revealed that poor differentiation is a risk indicator for lymphatic metastasis.

**Fig. 2:**
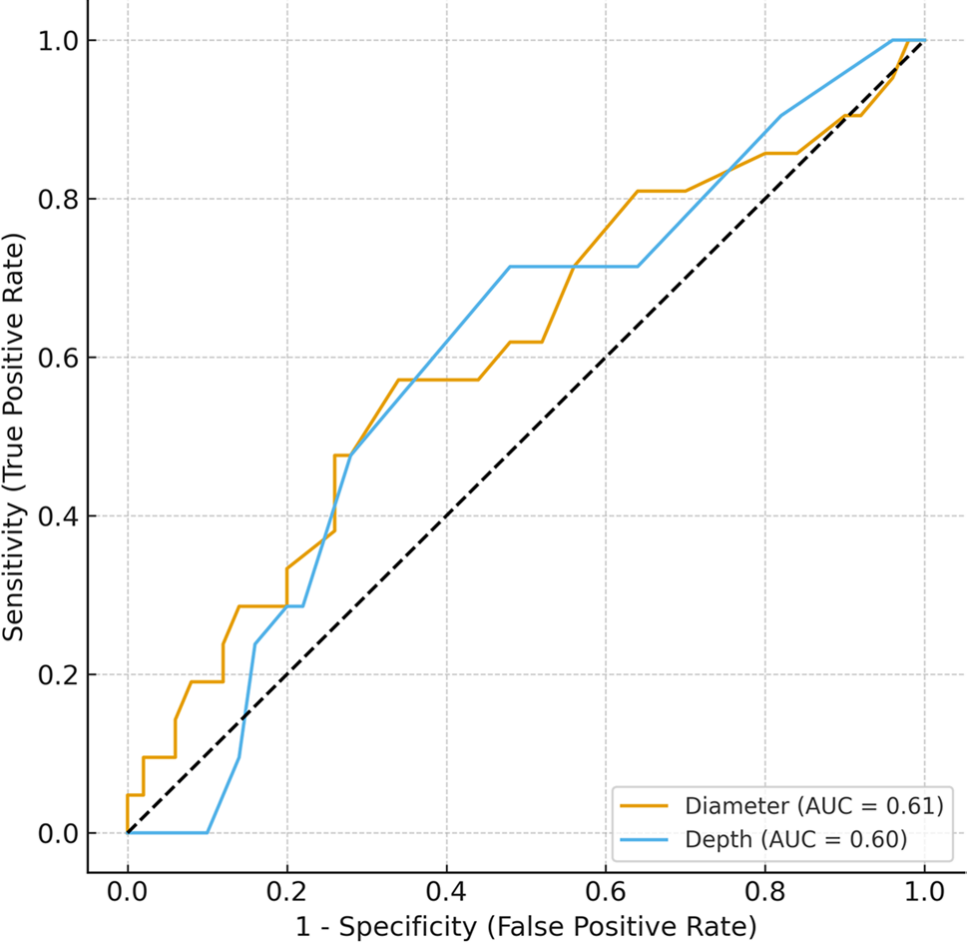
ROC Curves for lymph node metastasis prediction

With Youden’s index by using the ROC analysis, the best matching cut-off value for tumor diameter was 1.6 cm. This means that a tumor diameter > 1.6 cm comes with a higher risk for lymph nodal spreading. The cut-off value for depth of invasion was defined as 0.85 cm, so a depth of invasion > 0.85 cm is associated with a higher risk for lymph nodal spreading (Tables 3 and 4). Figure 2 shows curves for tumour diameter and depth of invasion.

A logistic regression model included the following parameters: G3 grading, cut-off-value for diameter and depth of invasion, R1 resection, desmoplastic type, basal-cell-carcinoma (BCC) combined with basal-cell-carcinoma (BCC) combined with CSCC and multiple CSCCs.

Poor differentiation (G3) was the greater strongest factor associated with lymph nodal spreading (*p* = 0.00). The odds ratio for developing lymph nodal spreading in G3 CSCC was 19.1, which means patients with G3 CSCC have a 19.-higher risk for developing metastases (Table 2). In a penalised bias-reduced logistic regression (Firth approximation), G3 grade remained strongly associated with lymph node metastasis (OR 19.7), while depth of invasion > 0.85 cm showed a weaker association (OR 1.75). These findings are consistent with the conventional and ridge-penalised models, supporting the robustness of G3 as the most influential risk indicator despite the low events-per-variable ratio.

**Table 2:** Logistic regression model for metatases

Parameter	*p*-value	95% Confidence intervall Lower value	95% Confidence intervall Higher value
Cut off diameter	0.770	0.092	5,869
Cut off depth of invasion	0.438	0.157	71,763
G3 (grading)	0.000	0.001	0.114
R-status	0.762	0.001	227,721
BCC + CSCC	0.440	0.002	13,712
Multiple CSCC	0.566	0.062	161,513
Desmoplastic type	0.989	0.001	638,279

All other considered parameters were not statistically significant, respectively. Tables 3 and 4 show the distribution of tumor diameter and metastases as well as the depth of invasion and metastases. According to the staging system, which differentiates T stages according to tumor thickness, we have calculated our patients’ specific risk for metastases. In cases of tumor thickness between 2 and 6 mm, the incidence of lymph node metastasis was 9.3%, while for all patients with thickness greater than 6 mm, it was 22.2%.

**Table 3:** Tumor diameter and appearance of metastases

Tumor diameter and metastases			
	metastases		All patients
	no metastases	metastases	
Diameter < 1.6 cm	24 (33.8%)	8 (11.3%)	32 (45.1%)
Diameter > 1.6 cm	26 (36.6%)	13 (18.3%)	39 (54.9%)
All patients	50 (70.4%)	21 (29.6%)	71 (100%)

**Table 4:** Depth of invasion and appearance of metastases

Depth of invasion (DOI) and metastases			
	metastase		All patients
	no metastase	metastase	
DOI < 0.85 cm	38 (53.5%)	12 (16.9%)	50 (70.4%)
DOI > 0.85 cm	15 (21.1%)	6 (8.5%)	21 (29.6%)
All patients	53 (74.6%)	18 (25.3%)	71 (100%)

#### Localization of metastases

The most affected level turned out to be level Ib (18 cases, 85.7%). Table 1 gives an overview of all patients suffering from lymph nodal spreading and the concerned levels.

In our study, lymph nodal spreading ranged from level Ia to combined levels up to level IV (Table 1).

## Discussion

While patients with facial CSCC can be treated successfully with surgical resection, lymph node metastases worsen the prognosis noticeably. Five-year survival rates in patients with proven lymph node metastases can drop down to 25% [13].

Although current literature considering all tumor sites located in the face and skull describes a rate of 5% of lymphatic metastases in patients with CSCC, our study observed a rate of 29.6% of all patients developing lymph nodal spreading during the follow-up time. A reason for the high incidence of metastases in the present patient group could be the presence of one or more of the following high-risk factors and the fact that all patients have been treated in a University Center. Therefore, some patients initially presented with large tumors and advanced stages. Also long time follow-up was well documented in our study. However, the lip as localization may play a role in higher risk for metastases. Dinehart et al. 1989 have already argued the lip as a site with increased metastatic potential, nevertheless studies considering only CSCC of the lip are rare so far [11]. Most studies combine oral squamous cell carcinomas and CSCC of the lip. This study aims to focus on patients suffering from lymph nodal spreading and risk factors for lymph nodal spreading in CSCC of the lower lip only. This discrepancy is likely multifactorial. Our cohort represents a preselected group of complex and advanced cases referred to a tertiary university hospital, often after prior incomplete (non-sano) excisions or delayed presentation. Such circumstances may have facilitated earlier metastatic spread. In addition, several patients already exhibited radiologically suspicious lymph nodes at initial staging, reflecting an inherently higher-risk population. Nevertheless, biological and anatomical factors of the lower lip—such as its rich lymphovascular network, thin mucocutaneous transition zone, and continuous UV exposure—may also contribute to a greater metastatic potential compared with other head and neck sites. All patients, including those aged over 85 years, were followed for at least 36 months or until death, ensuring robust detection of late metastatic events.

In this study only patients with CSCC of the lip were included. Amin et al. [14] argued tumor diameter > 2 cm is the risk factor most associated with disease-specific death, with a 3-fold higher risk of recurrence and a 6-fold higher risk of metastasis [14]. In our study the cut-off value regarding tumor diameter for higher risk of developing metastases was 1.6 cm, which is slightly lower than in the TNM classification. On the other hand, Thompson et al. [10] reported in a meta-analysis the optimal cut-off value is 4 cm for determining the risk for metastasis and local recurrence [10]. In the present study, there was no correlation between tumor diameter and lymph node metastasis using logistic regression analysis. Also, separate statistical analysis using the chi-square test did not show any significance in our data.

Tumor thickness is known to be an important predictor for nodal spread in patients with CSCC. In a prospective investigation of 615 patients performed by Brantsch et al. [6] the incidence of lymph node metastasis was 0% in patients with tumor thickness of less than 2 mm [6, 15]. This is in line with our findings. In cases of tumor thickness between 2 mm and 6 mm the incidence for developing lymph node metastasis was 9.3% and 22.2% for patients with tumor thickness greater than 6 mm in our study. Those results are slightly higher than those published in the literature [6], possibly due to our patient group with further progressed stages when initially presenting at the university hospital [6]. Other studies often evaluated only CSCC in general and did not consider isolated localizations like the lip. The cut-off value for depth of invasion was reported to be 0.85 cm in our data.

R1 tumor resection (involved margins) is associated with a higher risk for local recurrence and lymph nodal spreading [16, 17]. This result can be confirmed by the present study. No statistically significant correlation between R1 resection and metastasis was observed; however, the analysis was limited by the small number of patients with R1 resection (n = 5), of whom 4 developed lymph node metastases. Although no significance can be described due to low patient numbers, a correlation can be assumed.

Poor differentiation is known to be a strong predictor for developing lymph node metastasis in patients with CSCC [18]. In the present study, 14 out of 18 patients suffering from lymph nodal spreading had a histologically proven poor differentiated CSCC with G3 being statistically significant (*p* = 0.00).

In the present study, the risk of developing lymph node metastasis in patients with G3 was 19.1 times higher. Obermeier et al. [18] reported a 14-times (5–10% commonly reported in literature) higher risk for developing lymph nodal spreading in CSCC for patients with G3 (poor differentiated) CSCC relating to all tumor localisations. Peat et al. [19] reported the incidence of nodal metastasis to be 37% in poor differentiated CSCC [19]. This discrepancy may be explained by the fact that our study focused exclusively on lower lip CSCC, a site associated with higher metastatic potential, and that as a tertiary referral university hospital we treated a high proportion of patients presenting with advanced stages and large tumors. Furthermore, the long-term follow-up in our cohort allowed for detection of nodal recurrences that may be underestimated in other series. Anyway, other histopathological parameters as immunohistochemical analysis with elevated Ki67 (cell expression), p53 or VEGF should be considered in the pathogenesis of lymph nodal spreading and metastases [20].

As shown in our results, the lower lip has a high risk for lymph nodal spreading in poor differentiated CSCCs.

## Conclusion

The risk for lymph nodal spreading in CSCC is low in general. Patients with extensive disease show an increased risk of lymphatic metastasis, as do patients suffering from CSCC of the lower lip. Different risk factors for lymph nodal spreading are reported in the literature, but in our patients with CSCC of the lower lip, mainly G3 grading (poor differentiated) was strongly associated with lymphatic metastases, in our cohort, with an odds ratio of 19.1. While this highlights tumor grade as a major risk indicator, further prospective studies are required to confirm causality and to account for additional biological and environmental factors.

## Data Availability

Not applicable.
